# Improved in-cell structure determination of proteins at near-physiological concentration

**DOI:** 10.1038/srep38312

**Published:** 2016-12-02

**Authors:** Teppei Ikeya, Tomomi Hanashima, Saori Hosoya, Manato Shimazaki, Shiro Ikeda, Masaki Mishima, Peter Güntert, Yutaka Ito

**Affiliations:** 1 Department of Chemistry, Graduate School of Science and Engineering, Tokyo Metropolitan University, Tokyo, 192-0397, Japan; 2 CREST/Japan Science and Technology Agency (JST), 4-1-8 Honcho, Kawaguchi, Saitama, 332-0012, Japan; 3 The Institute of Statistical Mathematics, 10-3 Midori-cho, Tachikawa, Tokyo, 190-8562, Japan; 4 Institute of Biophysical Chemistry, Center for Biomolecular Magnetic Resonance, Goethe University Frankfurt, 60438, Frankfurt am Main, Germany; 5 Laboratory of Physical Chemistry, ETH Zürich, 8093, Zurich, Switzerland

## Abstract

**I**nvestigating three-dimensional (3D) structures of proteins in living cells by in-cell nuclear magnetic resonance (NMR) spectroscopy opens an avenue towards understanding the structural basis of their functions and physical properties under physiological conditions inside cells. In-cell NMR provides data at atomic resolution non-invasively, and has been used to detect protein-protein interactions, thermodynamics of protein stability, the behavior of intrinsically disordered proteins, etc. in cells. However, so far only a single *de novo* 3D protein structure could be determined based on data derived only from in-cell NMR. Here we introduce methods that enable in-cell NMR protein structure determination for a larger number of proteins at concentrations that approach physiological ones. The new methods comprise (1) advances in the processing of non-uniformly sampled NMR data, which reduces the measurement time for the intrinsically short-lived in-cell NMR samples, (2) automatic chemical shift assignment for obtaining an optimal resonance assignment, and (3) structure refinement with Bayesian inference, which makes it possible to calculate accurate 3D protein structures from sparse data sets of conformational restraints. As an example application we determined the structure of the B1 domain of protein G at about 250 μM concentration in living *E. coli* cells.

In-cell NMR spectroscopy was first proposed as a tool for investigating the behavior of bio-macromolecules at high resolution in living cells^1^. Extending this method to protein structure determination, we obtained the first three-dimensional (3D) protein structure calculated exclusively on the basis of experimental data measured from living *Escherichia coli* (*E. coli*) cells for the putative heavy-metal binding protein TTHA1718 from *Thermus thermophilus* HB8^2^,^3^. In comparison with conventional *in vitro* protein structure determination approaches by X-ray crystallography, NMR, or electron microscopy, this approach has unique abilities that permit to investigate the effect of macromolecular crowding^4^ and specific interactions among molecules in the 3D structures of proteins at work in their natural environment. Despite the high interest in studying protein conformations with atomic resolution in cells, however, to the best of our knowledge, additional in-cell structure determinations have not been presented since our report^2^ in 2009. Presumably, the method is still not robust or versatile enough, or too laborious for routine application to various proteins and associated functional studies. It is thus important to further improve the procedures for in-cell protein structure determination so as to make this approach more generally applicable.

Here, we present the methodological advances that were necessary to achieve a protein structure determination in living *E. coli* cells at an order of magnitude lower concentration than before. The structure of the *Streptococcus* protein G B1 domain consisting of 57 amino acids (henceforth referred to as GB1) in living *E. coli* cells was solved by in-cell NMR at a concentration of approximately 250 μM in the NMR tubes (Fig. S1), whereas in our previous publication^2^ the structure determination of the protein TTHA1718 required a concentration of 3–4 mM. Considering that the maximal natural concentration of a protein in normal cells is a few dozen to hundreds of μM^5^,^6^, our GB1 in-cell NMR samples approach the conditions of a physiologically natural environment. This result was achieved mainly by methodological advances in the three areas of NMR data processing of nonlinearly sampled data, automated chemical shift assignment, and robust structure calculation with Bayesian inference that can make optimal use of the limited experimental information (Fig. 1). For the NMR data processing of indirect dimensions of 3D NMR spectra^7^,^8^,^9^,^10^,^11^,^12^, we used the Quantitative Maximum Entropy (QME) method^13^ instead of the conventional 2D maximum entropy approach (MaxEnt)^14^ implemented in the program Azara^15^ that had been used for the previous structure determination of TTHA1718 in *E. coli* cells^2^. Chemical shifts of in-cell GB1 were assigned by combining conventional manual analysis with an automated assignment procedure using the FLYA algorithm^16^, which has recently been shown to enable automated structure determination exclusively from NOESY-type NMR spectra without input chemical shift assignments^17^,^18^. Using NOESY spectra was crucial for obtaining side-chain assignments because proteins in cells exhibit faster transverse relaxation that makes it in general impossible to collect a sufficient number of signals from through-bond spectra for side-chain resonance assignments, e.g. H(CCCO)NH. NOESY spectra, on the other hand, included a considerable number of signals from the side-chains. Although it is usually not trivial to determine resonance assignments from NOESY spectra by conventional manual analysis, the automated chemical shift assignment algorithm FLYA permitted to comprehensively analyze all spectra and to objectively validate the obscure resonances from the manual approach. Structure calculations were performed employing the program CYANA^19^,^20^ with the newly developed CYBAY (CYANA Bayesian inference) module, which is able to extract a maximum of structural information from the limited and ambiguous experimental NOESY data with much broader line shapes and low sensitivity that is available for proteins in cells.

## Results

### Preparation of GB1 in-cell NMR samples

The conditions for sample preparation, host *E. coli* strains, incubation temperature and duration, etc., and the probe temperature for NMR measurements of GB1 in *E. coli* cells were optimized so as to maximize the viability of cells and minimize the leakage of expressed GB1 into the medium. The subsequent NMR measurements were performed at 22 °C where GB1 was stable in the in-cell samples for at least 6 hours of NMR measurements, and the contribution from extracellular proteins was negligible (Fig. S2).

### NMR measurements and spectral processing for GB1 in-cell NMR samples

Seven 3D triple-resonance NMR spectra, HNCA, HN(CO)CA, HNCO, HN(CA)CO, CBCA(CO)NH, CBCANH, and HCACO, were measured for the backbone ^1^H/^13^C/^15^N resonance assignment of GB1 in *E. coli* cells. For the side-chain ^1^H/^13^C resonance assignment, 3D HBHA(CBCACO)NH, H(CCCO)NH, (H)CC(CO)NH, HCCH-COSY and HCCH-TOCSY spectra were measured. For the collection of NOE-derived distance restraints, three types of 3D NOESY spectra, ^15^N-separated, ^13^C-separated and ^13^C/^13^C-separated NOESYs, were measured. Methyl-selectively ^1^H/^13^C-labeled samples were used for the 3D ^13^C/^13^C-separated NOESY experiments. A nonlinear sampling scheme for the indirectly acquired dimensions was employed in order to overcome the problems of low sensitivity and short life times of the in-cell NMR samples (Table S1).

**Figure 2:**
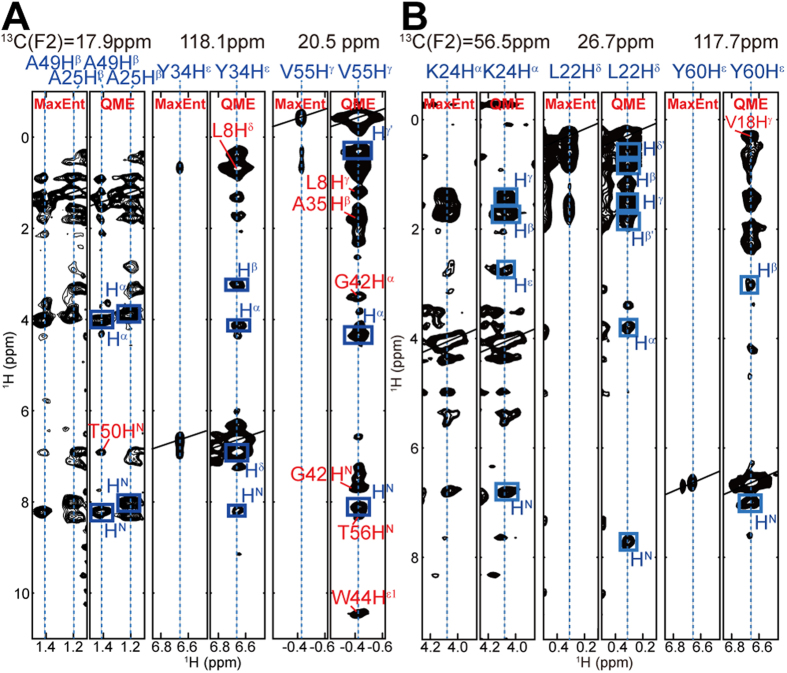
Comparison of 3D NOESY spectra of GB1 and TTHA1718 in *E.* *coli* cells processed with QME or MaxEnt reconstruction. *F*_1_(^1^H)-*F*_3_(^1^H) slices are shown from 2D MaxEnt and 2D QME reconstructed spectra for which the raw data were acquired using a nonlinear sampling scheme. (**A**) ^13^C-separated NOESY slices of GB1 at ^13^C frequencies of 17.9, 118.1, and 20.5 ppm. NOE-derived cross peaks that were undetectable by MaxEnt became visible at the ^13^C frequencies of 20.5 and 118.1 ppm in the QME-reconstructed spectra. (**B**) ^13^C-separated NOESY spectra of TTHA1718 at ^13^C frequencies of 56.5, 26.7 and 117.7 ppm. Undetectable NOE-derived cross peaks by MaxEnt were clearly observed for slices corresponding to the ^13^C frequencies of 26.7 and 117.7 ppm. Plotting parameters were kept identical within each 3D spectrum.

The low concentration of GB1 in *E. coli* cells compared to the former case^2^ of TTHA1718 resulted in a much-reduced contrast between NMR signals of GB1 and background. We previously applied QME to 3D NMR spectra of proteins in living sf9 cells, where the existence of strong and sometimes very sharp background signals due to endogenous and baculovirus-derived molecules was problematic^13^. Since expecting a similar or even more severe problem in HCCH- and NOESY-type spectra of GB1 in *E. coli* cells, in which strong self-correlated diagonal signals and plenty of much weaker correlation cross peaks are observed all together, we examined the reproducibility of the QME processing on reconstructing these spectra. Maximum entropy (MaxEnt) is a widely used method for NMR signal reconstruction that aims at minimizing an objective function, *Q*(*h*) = *λS*(*h*) – *L*(*h*), where *h* is mock data, *S* and *L* are the entropy and residual terms, respectively, and the Lagrange multiplier *λ* reflects the relative contribution of prior information based on the maximum entropy principle and the error of the experimental data. It is not trivial to manually determine the optimal value of *λ* based on general criteria. Whereas the conventional MaxEnt approach in the Azara software fixes *λ* to a used-defined value, QME chooses *λ* for an entire spectrum by an iterative search procedure for the maximum of an approximated conditional probability distribution for the experimental data given *λ*. QME improved drastically the quality of in-cell NMR spectra, which suffer from extraordinarily strong background signals from endogenously expressed proteins and a wide dynamic range in peak intensity. The comparison of spectra processed by QME and Azara MaxEnt indicated the superiority of the QME spectra in which the intensities of many cross peaks were clearly enhanced and cross peaks that were undetectable by MaxEnt became visible (Figs 2 and S3). Indeed, the number of picked NOESY cross peaks was significantly increased with QME processing, resulting in better side-chain assignments and 3D structures. Artificial peaks attributed to nonlinear sampling and QME reconstruction can be removed by considering the consistency and redundancy of target protein-derived peaks among spectra in the stages of peak picking, manual and automated resonance assignment, or ‘network anchoring’ function of CYANA^21^,^22^.

### Backbone and side-chain resonance assignments of GB1 in *E. coli* cells

Most in-cell NMR studies reported so far utilized resonance assignments that were transferred from those obtained *in vitro*. However, when analyzing proteins potentially experiencing conformational changes in the intracellular environment, an assignment process based exclusively on the in-cell NMR spectra is needed for an accurate and detailed interpretation of the NMR data because chemical shift changes may occur, which would hinder the transfer of *in vitro* assignments to in-cell spectra.

By employing the manual approach that had been used for the case of TTHA1718 in *E. coli* cells^2^, virtually complete backbone resonance assignments were achieved for GB1 in *E. coli* cells (Figs S4 and S5). In contrast, for the side-chain resonance assignment the conventional triple resonance experiments HBHA(CBCACO)NH, H(CCCO)NH, and (H)CC(CO)NH lacked many of the expected cross peaks for GB1 in *E. coli* cells. We therefore measured a 3D HCACO spectrum for the additional assignment of ^1^H^α^ resonances, and 3D HCCH-COSY and HCCH-TOCSY spectra for the side-chain resonance assignment, which had not been measured in the case of TTHA1718. An example of the assignment process is shown in Fig. S6. Since assignments of side chain methyl groups are crucial for the structure calculation, we performed NMR measurements of GB1 in *E. coli* cells with selectively ^1^H/^13^C-labeled methyl groups of Ala, Leu, Ile, and Val and thus could assign the ^1^H and ^13^C resonances of 15 out of 17 of these methyls (Fig. S4). Overall, the chemical shifts of 88% of ^1^H^α^, 71% of ^1^H^β^, and 32% of the other aliphatic ^1^H/^13^C side-chain resonances of GB1 in *E. coli* cells were assigned manually (Fig. S7).

The ^1^H/^13^C chemical shifts of additional side-chain resonances were assigned with the help of an automated approach based on NOESY spectra as well as the spectra used for the manual side-chain resonance assignments. We employed the FLYA automated assignment algorithm, which has previously been shown to provide assignments of *in vitro* spectra without requiring a specific set of spectra for the sequential assignment^17^,^18^. While it was impossible to obtain a sufficient number of signals from the triple resonance spectra for side-chain resonance assignments due to fast transverse relaxation in cells, NOESY spectra included signals presumably originating from side-chains. Although it is usually not trivial to achieve the assignment from NOESY by the manual approach because the very large number of NOE-based assignment possibilities cannot be checked exhaustively, the FLYA algorithm permitted to comprehensively analyze all spectra and to objectively assign resonances whose assignment had remained obscure in the manual approach. FLYA assigned thus additionally 48 ^1^H, 1 ^15^N, and 52 ^13^C resonances (Table S2).

The protein GB1 is known to have a very stable structure. The molecular crowding in cells results in only small changes of the backbone chemical shifts when compared with GB1 *in vitro* (Fig. S8). This suggests a close similarity between the in-cell and *in vitro* structures of GB1, and the latter can be used as a reference for validating the in-cell structure determination method. Details of the correlation of the chemical shift changes with the structures will be discussed below.

### Structure determination of GB1 in *E. coli* cells with automated NOESY cross peak assignment and Bayesian inference-assisted structure refinement

**Figure 3:**
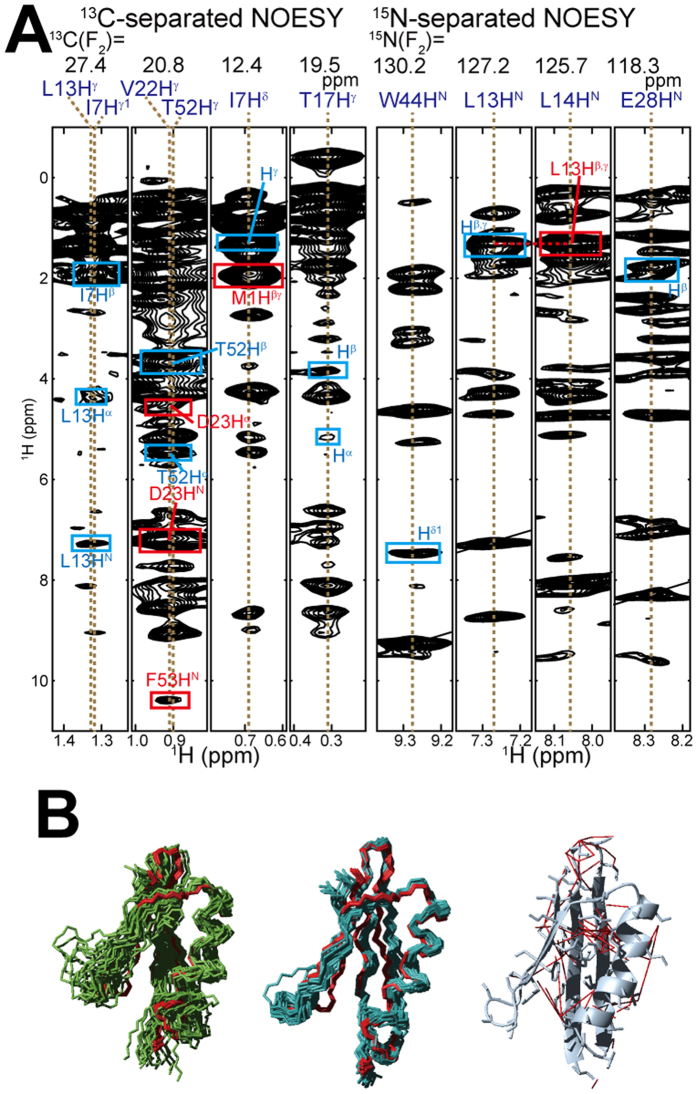
Automatically assigned chemical shifts by FLYA for GB1 in cells. (**A**) ^1^H-^1^H cross-sections corresponding to the ^13^C and ^15^N frequencies extracted from the ^13^C-separated and ^15^N-separated NOESY spectra. The atoms written on the spectra show NOE peaks and assignments that were additionally determined by the FLYA automatic analysis. In the ^13^C-separated NOESY the direct and indirect dimensions were assigned by FLYA, whereas in the ^15^N-separated NOESY only the indirect dimension was assigned by FLYA. Intra- and inter-residual NOEs are indicated by blue and red boxes, respectively. (**B**) Superposition of the 20 structures of GB1 in living *E. coli* cells without (green) and with (light blue) automatically assigned chemical shifts by FLYA, and *in vitro* (red), showing the backbone (N, C^α^, C’) atoms. Distance restraints derived on the basis of the automatically assigned chemical shifts are represented in the white ribbon model with red lines (right).

Overall, 390 NOE-derived distance restraints, including 108 long-range restraints, could be obtained from 3D ^15^N-separated NOESY, ^13^C-separated NOESY, and ^13^C/^13^C-separated NOESY with selective ^1^H/^13^C-labeling of the methyl groups, and were used in the structure calculation (Fig. 3A). The additional distance restraints that could be assigned with the chemical shifts from FLYA resulted in a structure that was clearly better defined and closer to the *in vitro* one. This indicates that the automatic chemical shift assignment based mainly on the NOESY spectra is effective for the structure determination particularly if one cannot obtain a sufficient number of signals from spectra for side-chain resonance assignment (Fig. 3B and Table S2).

Conventional NMR structure calculation consists essentially of a conformational search with simulated annealing (SA) by molecular dynamics simulation (MD), which aims at satisfying ranges of distances and dihedral angles derived from experimental data, and subsequent structure optimization in a physical force field. However, this approach did not perform adequately with the in-cell data on account of the sparse experimental structural information and limited conformational search range of SA. A more sophisticated method with a larger radius of convergence is needed in order to accurately evaluate the sparse and ambiguous experimental data derived from proteins in living cells. Thus, we adopted another NMR structure optimization method based on a Bayesian framework, so called Inferential Structure Determination (ISD)^23^. The Bayesian approach interprets the experimental data with the prior information including the physical force field, and conformations and explanatory variables of the data are searched extensively by using replica-exchange Monte Carlo (REXMC)^24^. Moreover, it yields the variables and final structure ensemble in the form of the posterior probability distribution, which enables us to validate the data and results statistically. While the original ISD approach has achieved considerable success^23^,^25^, it was not yet sufficient for the present in-cell NMR structure determination due to high background content and low signal-to-noise (S/N) ratio in the spectra, which severely limited the number of distance restraints that could be derived from the spectra. As a method for efficiently analyzing these in-cell NMR data along with the more sophisticated treatment of prior information, we developed, within the framework of the CYANA software package^19^,^20^, the CYBAY algorithm that is composed of automatic NOESY cross peak analysis^21^,^22^, fast global conformational search by torsion angle MD (TAMD) and structure optimization by REXMC with the physical force field. Recently, CYANA was also equipped with the Amber ff03 physical force field^26^ and a Generalized Born (GB) implicit water model^27^ that enable to search conformations more accurately on the energy landscape of proteins in torsion angle space, yielding more detailed prior distributions. The parameters of the force field and GB implicit water model were set to the standard values used in MD simulations with general water solvent, e.g. the dielectric constant of the solvent is 78.5, which may be slightly different under physiological crowding conditions inside cells. However, the force field with the water model is only used as prior information, and the posterior is updated based on the experimental data during the calculation. Thus, it is not necessary to employ (unknown) optimized solvent parameters for the cell environments in the calculations. TAMD in CYANA permits longer time steps than Cartesian space MD simulation, and thus provides a faster and wider conformational search. Moreover, CYBAY handles ambiguous NOE assignments in the calculation. As a result, CYBAY achieved more accurate and data-driven structure determination with the relatively poor in-cell NMR signals. A detailed presentation of CYBAY and its application to *in vitro* data of several proteins including a comparison to the conventional method have been published recently^28^. Here we show the application of CYBAY to in-cell NMR structure determination.

**Figure 4:**
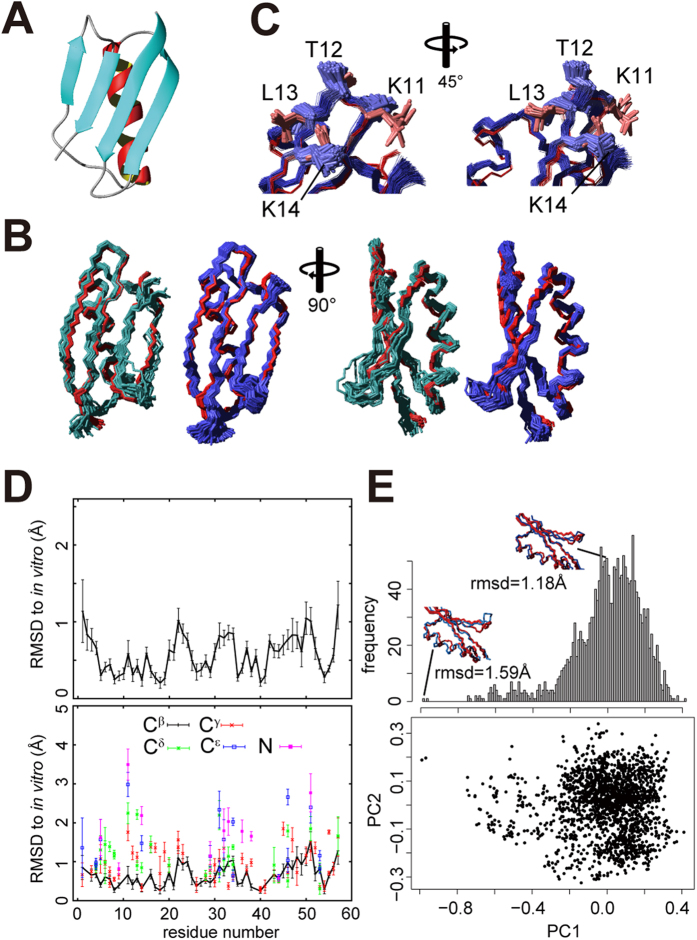
NMR structure of the protein GB1 in living *E. coli* cells. (**A**) Ribbon diagram of the structure with the highest posterior. (**B**) In-cell GB1 structures obtained by CYBAY (blue) and conventional CYANA calculation with the FLYA automatic chemical shift assignment (light blue), showing the backbone (N, C^α^, C’) atoms. 380 (20%) out of 1900 CYBAY conformers and 20 out of 100 in the final step of the conventional method are superimposed to the 20 structures determined *in vitro* (red), respectively. (**C**) Superpositions of the 20 GB1 structures determined *in vitro* (red) and the ensemble of in-cell CYBAY structures (blue), showing the side-chains of residues 11–14. (**D**) RMSD per residue and its standard deviation of the 1900 conformations for the backbone (N, C^α^, C’; top) and side-chain (C^β^, C^γ^, C^δ^, C^ε^ and N atoms; bottom). (**E**) Distributions of the first principal component (top) and the first and second ones (bottom).

The CYBAY calculation was performed with a sufficient 10^7^ REXMC steps. It converged well (Figs S9A and B), as indicated by the facts that exchanges among the 10 different runs (replicas) occurred often at all temperatures used, and that scores were on average stationary (Materials & Methods). The final 1900 conformers were selected from the MC sampling region in which the posterior was stationary (Fig. S9B). Figure 4A shows the representative CYBAY-refined structure with maximal a posteriori estimation (MAP) of GB1 in living cells. Meanwhile, one of the advantages of Bayesian inference is that it provides not only the best structure with the lowest target function value (or MAP), but also distributions that reflect the uncertainty of experimental data such as measurement errors and shortage of information. Figure S9C and D show unimodal, approximately normal distributions, indicating that the structure ensemble derived from the data did not provide multiple conformations along the axis of the physical potential energy. In order to analyze the structures based on representative variances of 3D coordinates, principal component analysis (PCA) was applied to the structure ensemble. Along the first principal component (PC1) a slightly non-normal distribution was observed (Fig. 4E), which suggests the presence of a small number of minor populations in the vicinity of the major region. To elucidate whether the minor populations are of physiological significance, additional NMR experiments such as CPMG relaxation dispersion^29^,^30^ will be needed. The distributions obtained by Bayesian inference allow to examine the probability of occurrence of conformations due to current data from various perspectives. This analysis based on the posterior distribution differs from a conventional structure determination, in which the 10 or 20 conformers with the lowest energies are selected to represent the final NMR structure. Whereas the major region comprises structures within about 1.2 Å RMSD from the *in vitro* structure determined independently by the conventional SA method, the minor populations include structures with approximately 1.6 Å RMSD (Fig. 4E). The CYBAY structure ensemble with 1900 conformers is well defined with an average backbone RMSD of 0.43 Å to the mean coordinates. The backbone RMSD between its mean structure and the *in vitro* structure is 1.18 Å. Alternatively, selecting the 20 highest posterior probability conformers (PDB accession code 2N9L) for comparison with the conventional method, the RMSD of these 20 structures is 0.49 Å to its mean and 1.02 Å to the *in vitro* structure (Table S2). Figure 4D shows the RMSD per residue to the *in vitro* structure and its standard deviation of all the sampled conformations. RMSDs of C^α^ atoms (upper panel of Fig. 4D) were below 1.0 Å for most residues, except for two loops of residues 22 and 50–51 that show slightly higher values around 1.0 Å. On the other hand, a loop and the end of a β-strand (residue 11–14) show low RMSDs to the *in vitro* structure for the C^α^ atoms but higher RMSDs of more than 2.0 Å for the side-chains (Fig. 4C and lower panel of 4D). These residues coincide well with a region of slightly higher chemical shift differences between the in-cell and *in vitro* samples (residue 10–13) (Fig. S8B and C). The slight structural changes of the side-chains may be due to molecular crowding effects or the intracellular environment. While it is likely that the side-chains interact with a particular endogenous molecule, there are possibilities of nonspecific charge-charge interactions. It is known that most proteins in *E.coli* are polyanions at physiological condition^31^. Considering that these residues are on the molecular surface and include two lysines, the interactions with other negatively charged molecules might result in the structural changes of side-chains. To elucidate this effect, *in vitro* experiments under artificial charged molecular crowders would be required.

In addition, Bayesian inference provided the distributions of the calibration constants and their standard deviations for the three NOESY spectra. These distributions (Fig. S9E and F) reflect the quality and quantity of the experimental data more directly than those of the structures. In particular, the distributions of the calibration constant and its standard deviation for the ^13^C/^13^C-separated NOESY were broader than for the ^15^N-separated and ^13^C-separated NOESYs, presumably due to the smaller number of peaks, and the concomitant smaller information content.

### In-cell structure of the protein TTHA1718

**Figure 5:**
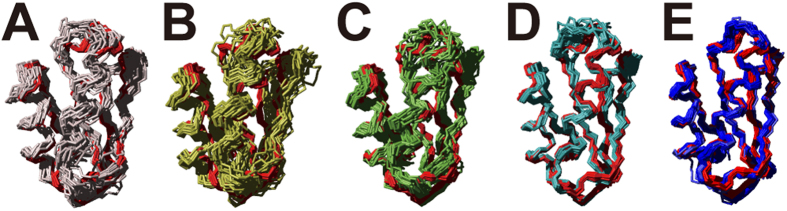
Structures of the protein TTHA1718 in living *E. coli* cells. (**A**) Previously reported structure of TTHA1718 in living *E. coli* cells^2^ computed with hydrogen bond restraints (grey). (**B**) Structure calculated as in *A*, but, as all the following structures, without hydrogen bond restraints (yellow). (**C**) Structure obtained with the NOESY spectra newly processed by QME (green). (**D**) Structure obtained using QME-processed spectra and additionally automatically assigned chemical shifts by FLYA (cyan). (**E**) Structure obtained using QME-processed spectra, FLYA automated assignments, and CYBAY Bayesian refinement (blue). For comparison, the structure determined *in vitro* is shown in red in all panels. All structures are represented by bundles of 20 CYANA conformers in *A*-*D* and 360 (20%) out of 1800 CYBAY conformers in *E*, showing the backbone (N, C^α^, C’) atoms.

In the case of the first in-cell structure determination^2^ of the protein TTHA1718, we employed backbone hydrogen bond restraints for the β-sheet and α-helix regions where their existence was indicated by NOEs. While this approach has been used also for *in vitro* NMR structure determinations, it may obscure deviations from canonical secondary structure manifested in the experimental data because it explicitly fixes standard secondary structure hydrogen bonds for ranges of residues identified by the spectroscopist. It is instructive to improve the TTHA1718 structures by our present approach of data-driven structure determination with prior information. Thus, QME data processing, FLYA automatic resonance assignment, and CYBAY Bayesian structure optimization were applied also to the previously recorded NMR data of TTHA1718 in living *E. coli* cells. Omitting the hydrogen bond restraints from the conventional in-cell structure determination approach^2^ resulted in a structure that is obviously different from the previously published^2^ one (Fig. 5A and B, and Table S3). The 3D ^13^C-separated, ^15^N-separated, and ^13^C/^13^C-separated NOESY spectra that had previously been processed by Azara MaxEnt were newly reconstructed by QME. As with the in-cell GB1 spectra, the QME reconstruction clearly enhanced the intensities of numerous cross peaks, and additional signals that had previously been obscured by noise could now be observed (Fig. 2B). FLYA automatic chemical shift assignment was performed with the NOESY spectra processed by QME as well as the other 3D triple-resonance spectra. FLYA additionally assigned 4 ^1^H and 60 ^13^C resonances (Table S3). Overall, 608 NOE-derived distance restraints, including 188 long-range restraints, could be obtained from 3D ^15^N-separated, ^13^C-separated, and ^13^C/^13^C-separated NOESY spectra, and were used in the structure calculation. The results (Fig. 5) show that the QME data processing, FLYA chemical shift assignment, and CYBAY structure refinement significantly improved the structures even without using hydrogen bond restraints. As in the case of GB1, PCA analysis showed a non-normal distribution along PC1, indicating that the ensemble includes one major and other minor populations in the vicinity of the major region (Fig. S10E). Previously, we reported^2^ structural differences in the flexible loop regions between the *in vitro* and the in-cell structures. The large deviation of the in-cell structure ensemble however prevented us from analyzing the structural differences in details (left panel of Fig. S11A). Using the present methods, the in-cell TTHA1718 structures were converged much better than before throughout the sequence (right panel of Fig. S11A), presumably due to the additional distance restraints identified by the FLYA analysis of the QME-processed NOESY spectra (Fig. S11B) and the improved distance accuracy by Bayesian inference. The new result validated that structure differences were indeed located in three dynamics loop regions (residue 9-12, 26-29 and 44-50), which may be affected by the viscosity and macromolecular crowding in the cytosol.

## Discussion

Our results demonstrate that three methodological advances in NMR data processing, automated assignment, and Bayesian structure determination, made it possible to determine a 3D protein structure by in-cell NMR with much lower protein concentrations in cells and without artificial restraints such as hydrogen bond information. The NMR data processing by QME clearly ameliorated the quality of the in-cell spectra in which the intensity of numerous cross peaks were enhanced and some were additionally observed. We employed it from some reconstruction methods in this study owing to its sufficient performance and convenience to the extent that we tested them with several *in vitro* data^32^. Considering present substantial progress in that field, on the other hand, it might be able to replace QME to other state-of-art reconstruction algorithms such as compressed sensing^33^,^34^. In contrast, it would be indispensable to employ the automatic resonance assignment by FLYA, and the structure calculation and optimization by CYBAY. Whereas various automatic chemical shift assignment algorithms have been reported so far, to the best of our knowledge, FLYA is only generally applicable approach that permits to assign the resonances exclusively on the basis of NOESY spectra. Since it is expected that in in-cell NMR studies signals needed for the assignment are observed only in the NOESY due to faster transverse relaxation of proteins in cells, it is necessary to address the NOESY-based assignment algorithm by FLYA. In the structure calculation, it was not sufficient to achieve accurate structures using the conventional method in terms of the limitation of the searching algorithm and lack of statistical data analysis. Thus, the CYBAY approach is also essential for our method.

The highest naturally occurring concentration of a protein in cells is estimated to reach hundreds of μM. The GB1 protein in our in-cell NMR samples had a concentration of approximately 250 μM, which thus approached physiologically natural conditions in a cell. It would be possible to determine in-cell protein structures in samples with even lower concentrations that are much closer to physiological concentration, e.g. by employing other selective isotope labeling techniques^35^ and the state-of-art reconstruction algorithms previously described, and suppressing cell death in the sample tube, thereby allowing NMR measurement times beyond 6 hours. The cell death suppression can be achieved using recent technology such as the Bioreactor system that continuously supplies fresh medium from outside the spectrometer^36^.

Recently, structure determinations of GB1 in *Xenopus laevis* oocyte were reported using exclusively pseudocontact shift and residual dipolar coupling data^37^,^38^. The protein structure analysis in eukaryotic cells is instructive regarding applications in drug discovery and medical science. However, those approaches still require the aid of statistical information derived from databases and modeling software such as Rosetta^39^, and principally obtain the structures without experimental data for the side-chains which are indispensable for functional analysis of proteins and drug design. On the other hand, our method is a data-driven *de novo* protein structure determination that can elucidate all-atom coordinates based on the sufficient number of experimental distance restraints derived from NOEs.

Moreover, our method is a general protocol that can be applied not only for in-cell structure determination but also with *in vitro* samples that are problematic due to low concentration, instability, higher molecular weight for NMR analysis, difficult sample purification, and so on. Our method permits to extend the range of applications of biomolecular NMR and to contribute to the investigation of protein conformations under various conditions at atomic resolution.

## Materials and Methods

### Sample preparation

The expression plasmid pET47b encoding the *Streptococcus* protein B G1 domain (GB1) gene was used for the protein expression. An additional glycine residue is inserted following the N-terminal methionine. In-cell NMR samples were prepared as follows. JM109 (DE3) *E. coli* cells harboring the GB1 expression plasmid were first grown in unlabeled M9 minimal medium. The production of uniformly ^13^C/^15^N-labeled GB1 was induced by the addition of isopropyl thio-β-D-thiogalactoside (Wako) to a final concentration of 0.5 mM following transfer of the bacteria into M9 minimal medium (100 ml) containing 2 g/l U-^13^C-glucose (Cambridge Isotope Laboratories) and 1 g/l ^15^NH_4_Cl (Cambridge Isotope Laboratories). For uniformly ^15^N-labeled or ^13^C-labeled samples, either U-^13^C-glucose or ^15^NH_4_Cl and were replaced with unlabeled compounds, respectively. For the production of GB1 samples with selectively protonated side-chain methyl groups of Ala, Ile, Leu and Val residues in a uniform ^2^H-background, protein expression was induced in 100% D_2_O M9 medium containing 2 g/l unlabeled glucose (Wako), 1 g/l ^15^NH_4_Cl, 100 mg/l [3-^13^C] alanine (ISOTEC) and 100 mg/l [3-methyl-^13^C, 3, 4, 4, 4-^2^H4] α-ketoisovalerate (Cambridge Isotope Laboratories). The incubation was continued for 3 hours at 22 °C. For ^13^C/^15^N-labeled and ^15^N-labeled samples, the cells were harvested by gentle centrifugation and placed as ~60% slurry with M9 medium containing 10% D_2_O (ISOTEC) into NMR tubes. The ^13^C-labeled and methyl-selectively ^1^H/^13^C-labeled samples were suspended with M9 medium containing ~100% D_2_O. The concentration of GB1 in *E. coli* samples was estimated by comparing the density of the Coomassie-stained bands in SDS-PAGE gels with those of the purified GB1. The stability of GB1 *E. coli* samples was monitored repeatedly by 2D ^1^H-^15^N HSQC spectra followed by plating colony tests^40^.

GB1 for *in vitro* NMR experiments was purified by IgG sepharose 6 Fast Flow affinity column chromatography (GE Healthcare) following cell lysis by sonication and high temperature (70 °C) treatment for 10 minutes. The final GB1 fractions were concentrated and dissolved in M9 medium containing 10% D_2_O for NMR experiments.

### NMR spectroscopy

NMR experiments were performed at 22 °C probe temperature in a triple-resonance cryoprobe fitted with a *z*-axis pulsed field gradient coil, using a Bruker AVANCE 600 MHz spectrometer. Backbone ^1^H^N^, ^15^N, ^13^C^α^, ^13^C’, and side-chain ^13^C^β^ resonance assignments of GB1 in living *E. coli* cells were performed by analyzing six 3D triple-resonance NMR spectra, HNCA, HN(CO)CA, CBCA(CO)NH, CBCANH, HNCO, and HN(CA)CO measured on ^13^C/^15^N-labeled samples, and an HCACO spectrum measured on a ^13^C-labeled sample. 3D HBHA(CBCACO)NH, H(CCCO)NH, (H)CC(CO)NH experiments on ^13^C/^15^N-labeled samples, and 3D HCCH-COSY and HCCH-TOCSY experiments on ^13^C-labeled samples were performed for side-chain ^1^H and ^13^C resonance assignments. A 15 ms ^13^C isotropic mixing time was employed for the (H)CC(CO)NH, H(CCCO)NH and HCCH-TOCSY experiments. Intraresidue and sequential NOEs involving methyl protons were also utilized for the assignment of Ala/Leu/Val/Ile methyl groups. For the collection of NOE-derived distance restraints, 3D ^15^N-separated and 3D ^13^C-separated NOESY-HSQC spectra were measured on uniformly ^15^N-labeled or ^13^C-labeled in-cell NMR samples, respectively. In addition, 3D ^13^C-separated NOESY-HSQC and 3D ^13^C/^13^C-separated HMQC-NOESY-HMQC spectra were measured on Ala/Leu/Val/Ile-methyl-selectively protonated samples. A 100 ms NOE mixing period was employed for the 3D NOESY experiments. All 2D and 3D NMR data were recorded using the States-TPPI protocol for quadrature detection in indirectly observed dimensions. Water flip-back ^1^H pulses and the WATERGATE pulse sequence were used for solvent suppression in the experiments performed on ^15^N-labeled and ^13^C/^15^N-labeled samples, whereas presaturation and gradient-spoil pulses were used for ^13^C-labeled and methyl-selectively ^1^H/^13^C-labeled samples. One protein sample was used to measure one 3D spectrum.

For all 3D NMR experiments, a nonlinear sampling scheme was utilized for the indirectly observed dimensions in order to reduce the measurement time (Table S1). For instance, for most of the backbone triple-resonance experiments, 264 complex points (25%) were selected in a pseudo-random fashion from the conventional regularly spaced grid of 48 (*t*_1_) x 22 (*t*_2_) sampling space. With a ~1 s recycle time (including acquisition) and eight scans par a FID, the duration of each 3D experiment was reduced to ~140 min. Similar approaches were used for the side-chain assignment spectra and the NOESY spectra (Table S1). To ensure that only data of intact samples were acquired, each 3D experiment was repeated several times interleaved with monitoring of the sample condition by a short 2D ^1^H-^15^N HSQC experiment. These 3D data were combined to generate a new data set with improved signal-to-noise ratio until the 2D spectra exhibited significant changes. Typically, two 3D data sets were combined.

### NMR data processing

NMR spectra were processed using the AZARA 2.7 software (W. Boucher, www.bio.cam.ac.uk/azara) and QME, and analyzed using an OpenGL version of the ANSIG 3.3 software and CcpNmr Analysis version 2.4.1^41^. QME was used for processing nonlinearly sampled ^13^C and ^15^N dimensions. In the analysis of TTHA1718, the identical data as reported previously^2^ were used. Exclusively 3D ^13^C-separated, ^15^N-separated and ^13^C/^13^C-separated NOESY data that had been processed by the Azara Maximum entropy (MaxEnt)^15^ in the previous report were newly reconstructed by QME. The Azara 2D MaxEnt was performed using default parameter values, except for the choice of noise level, and took 28 seconds on 1 core of a 2.8 GHz Intel XEON CPU. The 2D QME reconstruction required no input parameters and estimated its own noise level. It took 100 minutes on the same computer using 12 CPU cores.

### FLYA automatic chemical shift assignment

The automatic chemical shift assignment was performed based on the chemical shifts assigned manually and a preliminary structure obtained by a conventional structure calculation with the manually assigned restraints. The manual assignments were fixed during the FLYA calculation. The tolerance for chemical shift matching was set at 0.04 ppm for ^1^H and 0.4 ppm for ^13^C and ^15^N. All recorded spectra were used for the automatic assignment process. Peaks were manually selected after automatic peak picking with AZARA version 2.7 for TTHA1718 and CcpNmr Analysis version 2.41 for GB1. In order to assign only feasible resonances, the resonances that were clearly not observed as peaks in the spectra were excluded from the automatic process.

### Structure calculation

GB1 and TTHA1718 structures were calculated with the program CYANA version 3.9 using automated NOE assignment^21^,^22^ and torsion angle dynamics^19^ for the structure calculation, which was started from 100 conformers with random torsion angle values. The standard CYANA simulated annealing schedule was applied with 10000 torsion angle dynamics steps. Backbone torsion angle restraints obtained from chemical shifts with the program TALOS+^42^ were added to the input for CYANA. Distance restraints for hydrogen bonds were not introduced.

### CYBAY refinement

A conformer with the lowest target function value in CYANA was used for the subsequent CYBAY refinement. For the CYBAY structure calculation, the replica exchange hybrid Monte Carlo method was implemented into CYANA, which consists of Markov chain Monte Carlo (MCMC) and molecular dynamics simulations (MD) with the Amber physical force field and generalized Born implicit water model. 10 replica Monte Carlo (MC) calculations with different temperatures of 300 to 400 K were performed with 10,000 replica transitions, each consisting of 10,000,000 MC steps for obtaining the conformational prior. The prior of the calibration constants of NOESYs and those standard deviations were described by normal distributions and inverse gamma distribution, respectively, and likelihood was designed by normal distribution. Due to the trace of step evolutions of posterior, ensemble conformations and parameters were sampled from a certain value-equilibrated step. The final ensemble conformations were obtained from trajectories at 300 K. Since the initial steps of the MC samplings were not stationary, the first 1,000,000 and 2,000,000 MC steps of the GB1 and TTHA1718 calculations, respectively, were excluded from the analysis. Selecting a conformer every 5000 MC steps, 1900 GB1 and 1800 TTHA1718 conformers were thus selected as the final structures. For the graphical representation of the structures, we randomly selected 20% of these final conformers.

## Additional Information

**How to cite this article**: Ikeya, T. *et al*. Improved in-cell structure determination of proteins at near-physiological concentration. *Sci. Rep.* **6**, 38312; doi: 10.1038/srep38312 (2016).

**Publisher’s note:** Springer Nature remains neutral with regard to jurisdictional claims in published maps and institutional affiliations.

## Supplementary Material

Supplementary Information

## Figures and Tables

**Figure 1:**
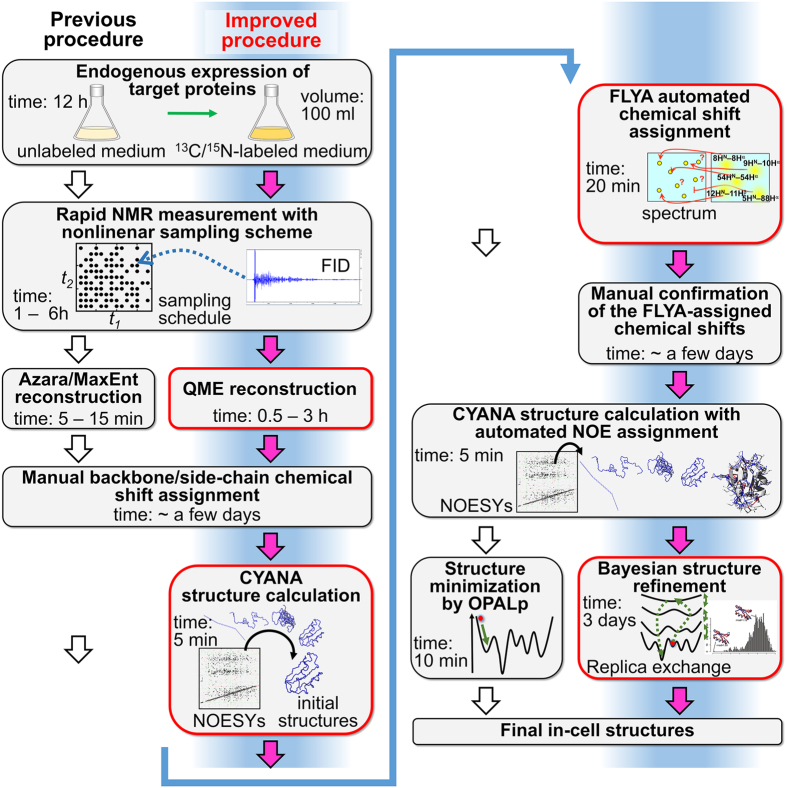
Side-by-side comparison of in-cell NMR protein structure determination by the previously proposed approach (2) and our improved procedure (blue). New and improved steps are shown in red boxes: QME data processing nonlinearly sampled data, FLYA automated chemical shift assignment, and CYANA structure calculation with Bayesian inference-based refinement to obtain the final in-cell structures. Approximate required times are indicated.
