# Supplementary material for: Improved in-cell structure determination of proteins at near-physiological concentration
Source: Sci Rep. 2016 Dec 2;6:38312. doi: 10.1038/srep38312 (PMC5133543; doi:10.1038/srep38312)
Supplement: Supplementary Information [file srep38312-s1.docx]

Supplementary Information for

Improved in-cell structure determination of proteins at
near-physiological concentration

**Teppei Ikeya^a,b,1^, Tomomi Hanashima^a^, Saori Hosoya^a^, Manato Shimazaki^a^,
Shiro Ikeda^c^, Masaki Mishima^a,b^, Peter Güntert^a,b,d,e^, and Yutaka Ito^a,b,1^**

^a^Department of Chemistry, Graduate School of Science and Engineering, Tokyo Metropolitan University, Tokyo 192-0397, Japan; ^b^CREST/Japan Science and Technology Agency (JST), 4-1-8 Honcho, Kawaguchi, Saitama 332-0012, Japan; ^c^The Institute of Statistical Mathematics, 10-3 Midori-cho, Tachikawa, Tokyo 190-8562, Japan; ^d^Institute of Biophysical Chemistry, Center for Biomolecular Magnetic Resonance, Goethe University Frankfurt, 60438 Frankfurt am Main, Germany; ^e^Laboratory of Physical Chemistry, ETH Zürich, 8093 Zurich, Switzerland.

^1^To whom correspondence should be addressed. E-mail: tikeya@tmu.ac.jp or
ito-yutaka@tmu.ac.jp.

Contents

**Supplementary Figure S1.** Estimation of the concentration of GB1 in *E. coli* cell NMR samples.

**Supplementary Figure S2.** Stability of *E. coli* cells expressing GB1 under NMR measurement conditions.

**Supplementary Figure S3.** Comparison of 3D NMR spectra of GB1 in E. coli cells processed with QME or MaxEnt reconstruction.

**Supplementary Figure S4.** Collection of nuclear Overhauser effect-derived distance restraints for the protein GB1 in living *E. coli* cells.

**Supplementary Figure S5.** Backbone resonance assignment of GB1 in *E. coli* cells.

**Supplementary Figure S6.** Side-chain resonance assignment of GB1 in *E. coli* cells.

**Supplementary Figure S7.** Backbone and side-chain resonance assignments of GB1 in living *E. coli* cells.

**Supplementary Figure S8.** Chemical shift differences of backbone ^1^H^N^ and ^15^N nuclei of GB1 between in *E. coli* cells and *in vitro* conditions.

**Supplementary Figure S9.** CYBAY statistics for GB1 in *E. coli* cells.

**Supplementary Figure S10.** CYBAY statistics forTTHA1718 in *E. coli* cells

**Supplementary Figure S11.** RMSDs for residues of TTHA1718 to the *in vitro* structures, and medium and long range distance restrains.

**Supplementary Table S1.** 3D NMR spectra measured for GB1 in living cells

**Supplementary Table S2.** NMR structure statistics of GB1 with in-cell NMR data

**Supplementary Table S3.** NMR structure statistics of TTHA1718 with in-cell NMR data


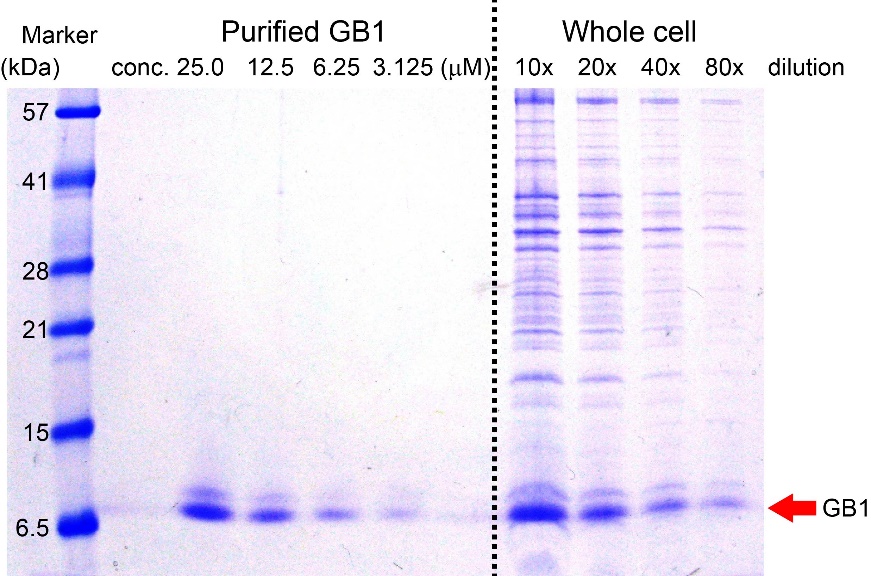


**Supplementary Figure S1. Estimation of the concentration of GB1 in *E. coli* cell NMR samples.** The concentration of GB1 expressed in *E. coli* NMR samples was estimated to be approximately 250 μM by comparing the density of the Coomassie-stained bands in SDS-PAGE gels with those of purified GB1, for which the concentration was determined independently.

**
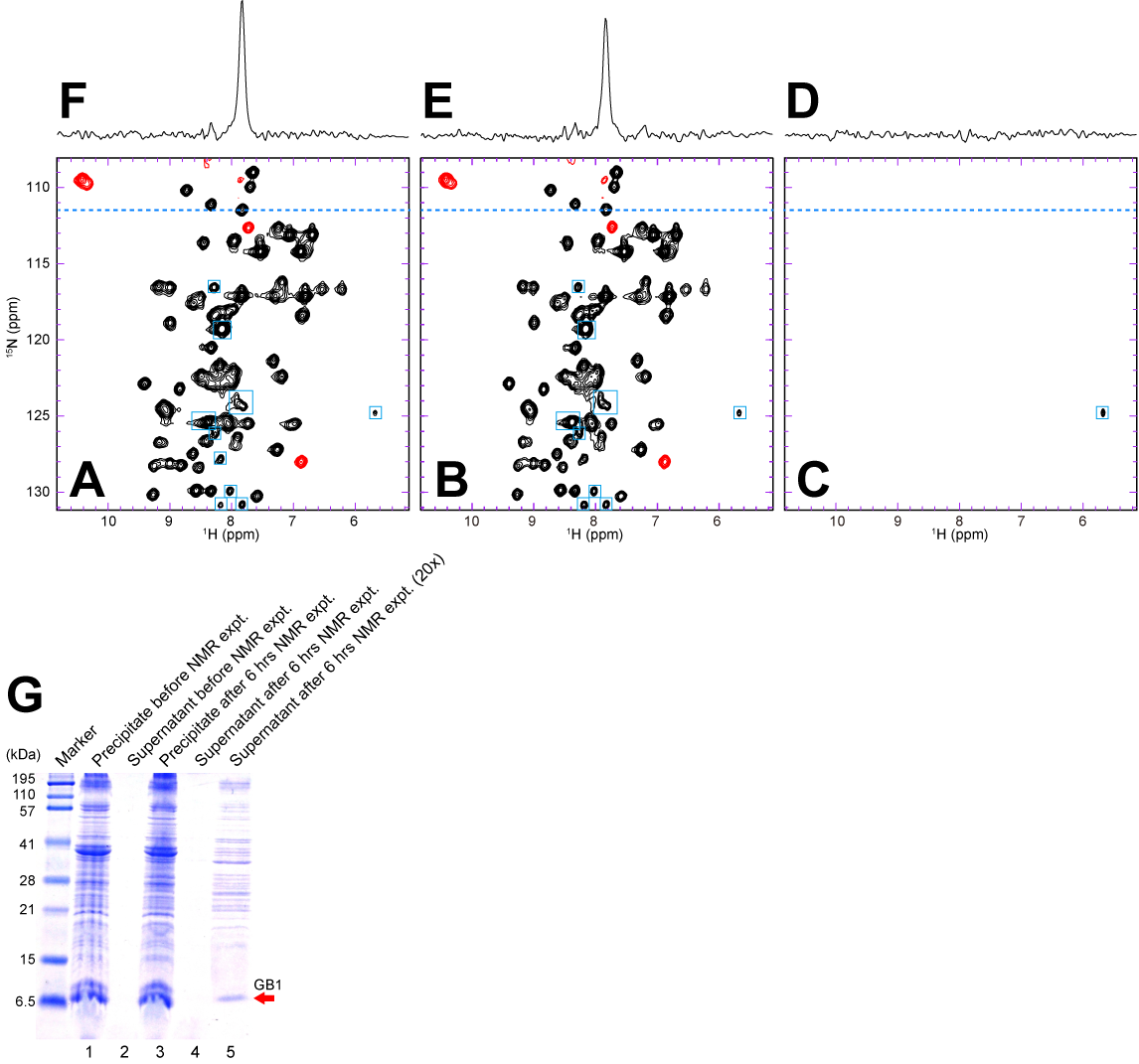
**

**Supplementary Figure S2.** **Stability of *E. coli* cells expressing GB1 under NMR measurement conditions.** 2D ^1^H-^15^N HSQC spectra of a GB1 in-cell NMR sample: (*A*) Immediately after sample preparation. (*B*) After 6 hours in an NMR tube at 22 °C. (*C*) Supernatant after 6 hours measurement, which is the in-cell NMR sample used in *A* and *B*. 1D cross sections taken at the position indicated by the dotted lines are shown above the corresponding 2D spectra (*D*, *E* and *F*, respectively). Light blue squares in *A*, *B*and *C* show background signals derived from intracellular compounds. ^1^H-^15^N HSQC spectra shown in *A***,** *B* and *C* were measured with 8 scans and a total of 512 (*t*_2_, ^1^H^N^) × 64 (*t*_1_, ^15^N) complex points. The measurement time was 20 min. (*G*) SDS-PAGE with Coomassie staining performed on in-cell NMR samples demonstrating that the proteins providing the NMR spectra in *A* and *B* (corresponding to lanes 1 and 3, respectively) are indeed inside the living cells and the contribution of extracellular protein to the observed signals is negligible.


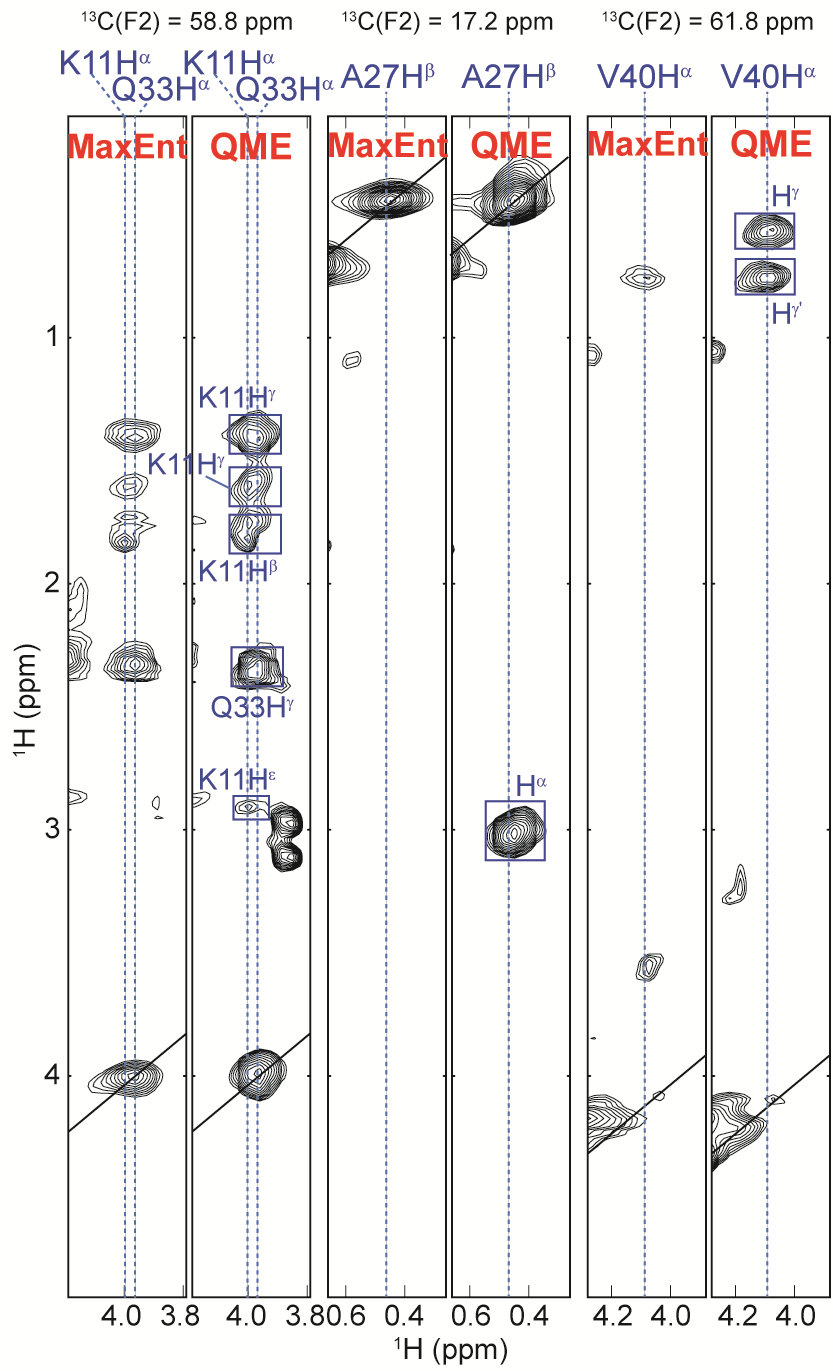


**Supplementary Figure S3. Comparison of 3D NMR spectra of GB1 in *E. coli* cells processed with QME or MaxEnt reconstruction.** *F*_1_(^1^H)-*F*_3_(^1^H) slices of 3D HCCH-TOCSY at ^13^C frequencies of 58.8, 17.2, and 61.8 ppm are shown from 2D MaxEnt and 2D QME reconstructed spectra for which the raw data were acquired using a non-linear sampling scheme. Cross peaks are clearly visible at the ^13^C frequency of 58.8 ppm, but largely absent for the other two ^13^C frequencies.


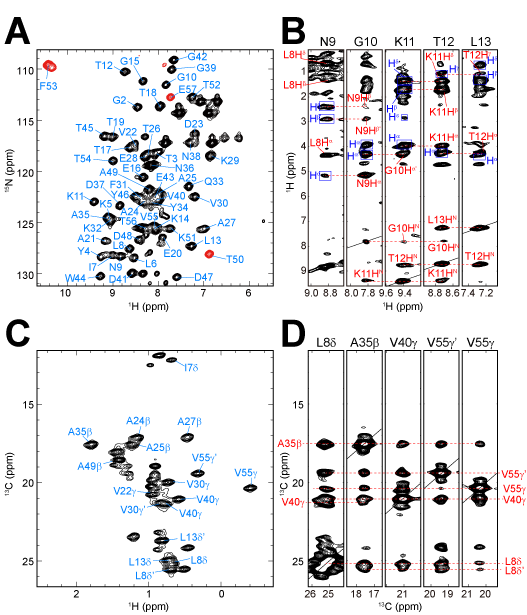


**Supplementary Figure S4.** **Collection of nuclear Overhauser effect-derived distance restraints for the protein GB1 in living *E. coli* cells.** (*A*) ^1^H-^15^N HSQC spectrum of a GB1 in-cell NMR sample. Cross peaks are labeled with their corresponding backbone assignments. (*B*) ^1^H-^1^H cross-sections corresponding to the ^15^N frequencies of selected backbone amide groups extracted from the 3D ^15^N-separated NOESY-HSQC spectrum. The cross peaks due to interresidual NOEs are assigned in red. Intraresidual NOEs are indicated by blue boxes and annotated. (*C*) Methyl region of the ^1^H-^13^C HMQC spectrum of the selectively methyl-protonated sample. Assignments of the methyl groups of Ala, Leu and Val residues are indicated, if available. Methyl cross peaks which were not assigned unambiguously are not annotated. Cross peaks from the precursors used for selective methyl protonation are also unlabeled. (*D*) ^13^C-^13^C cross-sections corresponding to the ^1^H frequencies of representative methyl groups extracted from the 3D ^13^C/^13^C-separated HMQC-NOESY-HMQC spectrum. Interresidual NOEs are indicated as in *B*. Using the precursor [3-methyl-^13^C, 3,4,4,4-^2^H4] α-ketoisovalerate, on the other hand, intraresidual NOEs were not observed in *D*.


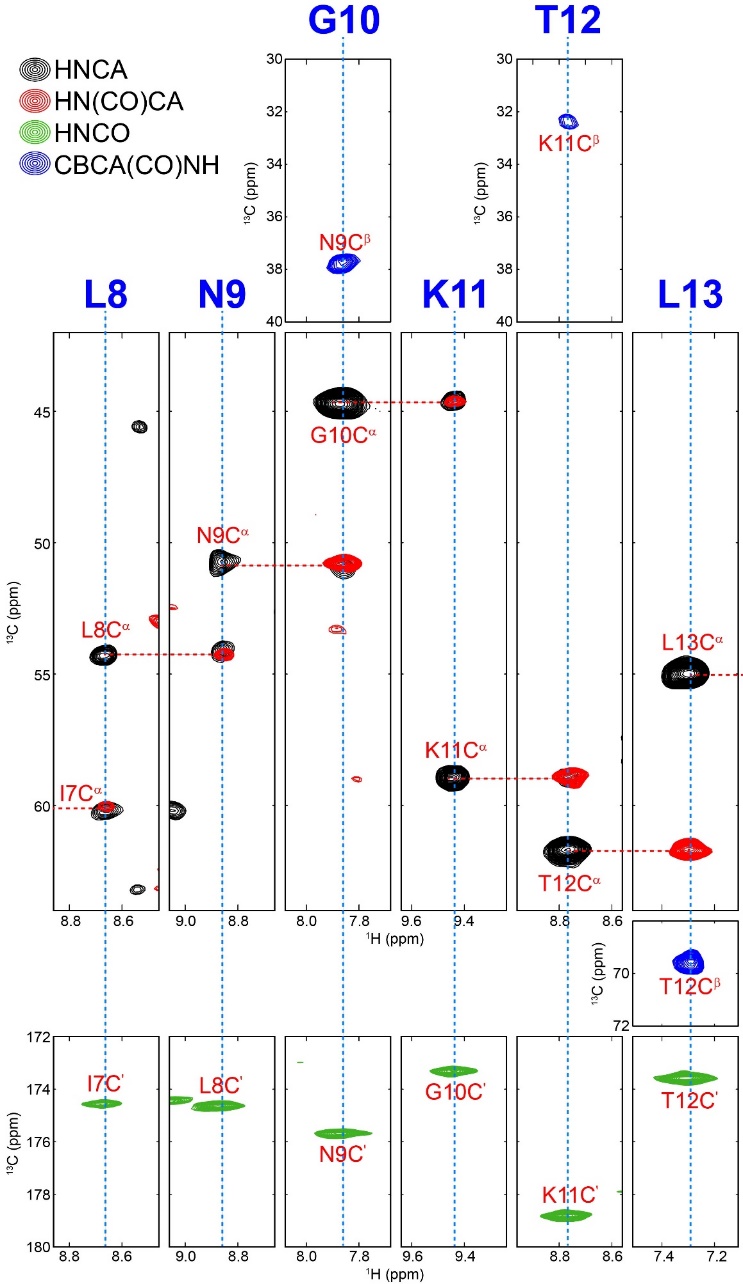


**Supplementary Figure S5. Backbone resonance assignment of GB1 in *E. coli* cells.**

Selected *F*_1_(^13^C)-*F*_3_(^1^H^N^) strips extracted from 3D HNCA (black), HN(CO)CA (red), 3D CBCA(CO)NH (blue), and 3D HNCO (green) spectra are shown. Each strip corresponds to the ^15^N frequency of the residue indicated. In the HNCA/HN(CO)CA spectra, sequential connectivities are represented by dashed red lines. Cross peaks are labeled with their assignments.


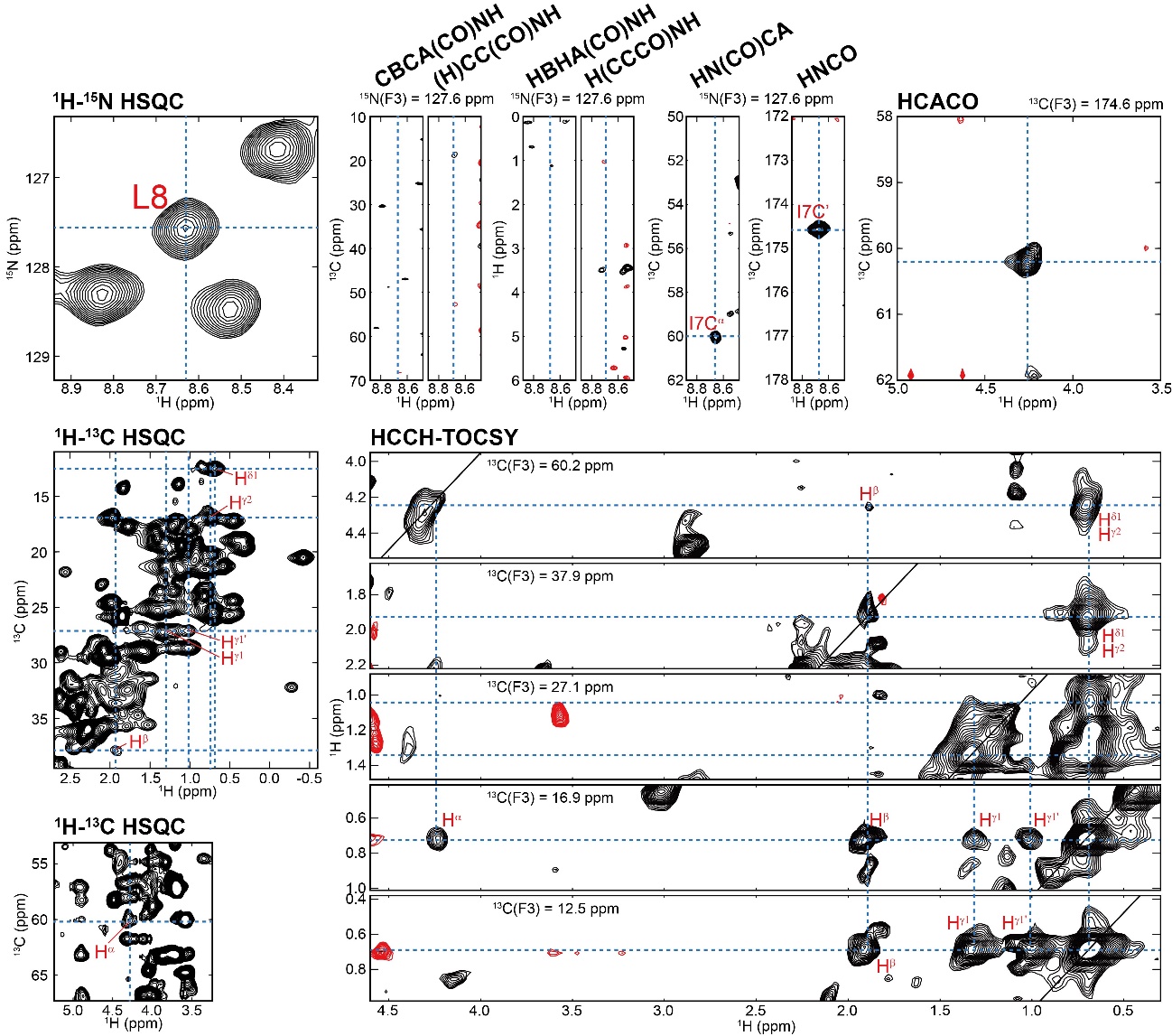


**Supplementary Figure S6. Side-chain resonance assignment of GB1 in *E. coli* cells.**

As an example, the manual assignment process for side-chain ^1^H/^13^C resonances of residue Ile 7 using exclusively 2D/3D in-cell NMR spectra is illustrated. The *F*_1_(^13^C)-*F*_3_(^1^H^N^) slices extracted from CBCA(CO)NH and (H)CC(CO)NH spectra and *F*_1_(^1^H)-*F*_3_(^1^H^N^) slices extracted from HBHA(CBCACO)NH and H(CCCO)NH spectra corresponding to the ^15^N frequency of Leu 8 (127.6 ppm) showed no cross-peaks. However, ^13^C^α^ and ^13^C’ resonances were observed in the HN(CO)CA and HNCO spectra, respectively, which were used for the assignment of the ^1^H^α^ resonance of Ile 7 in the *F*_1_(^13^C)-*F*_3_(^1^H) slice of the HCACO spectrum corresponding to the ^13^C’ frequency of Ile 7 (174.6 ppm). Starting from the ^1^H^α^ and ^13^C^α^ resonance assignments intraresidual connectivities were searched in the slices from the HCCH-TOCSY spectrum. Eventually, all side-chain ^1^H/^13^C resonances of Ile 7 could be assigned. The assignments are shown in the 2D ^1^H-^13^C HSQC spectrum.


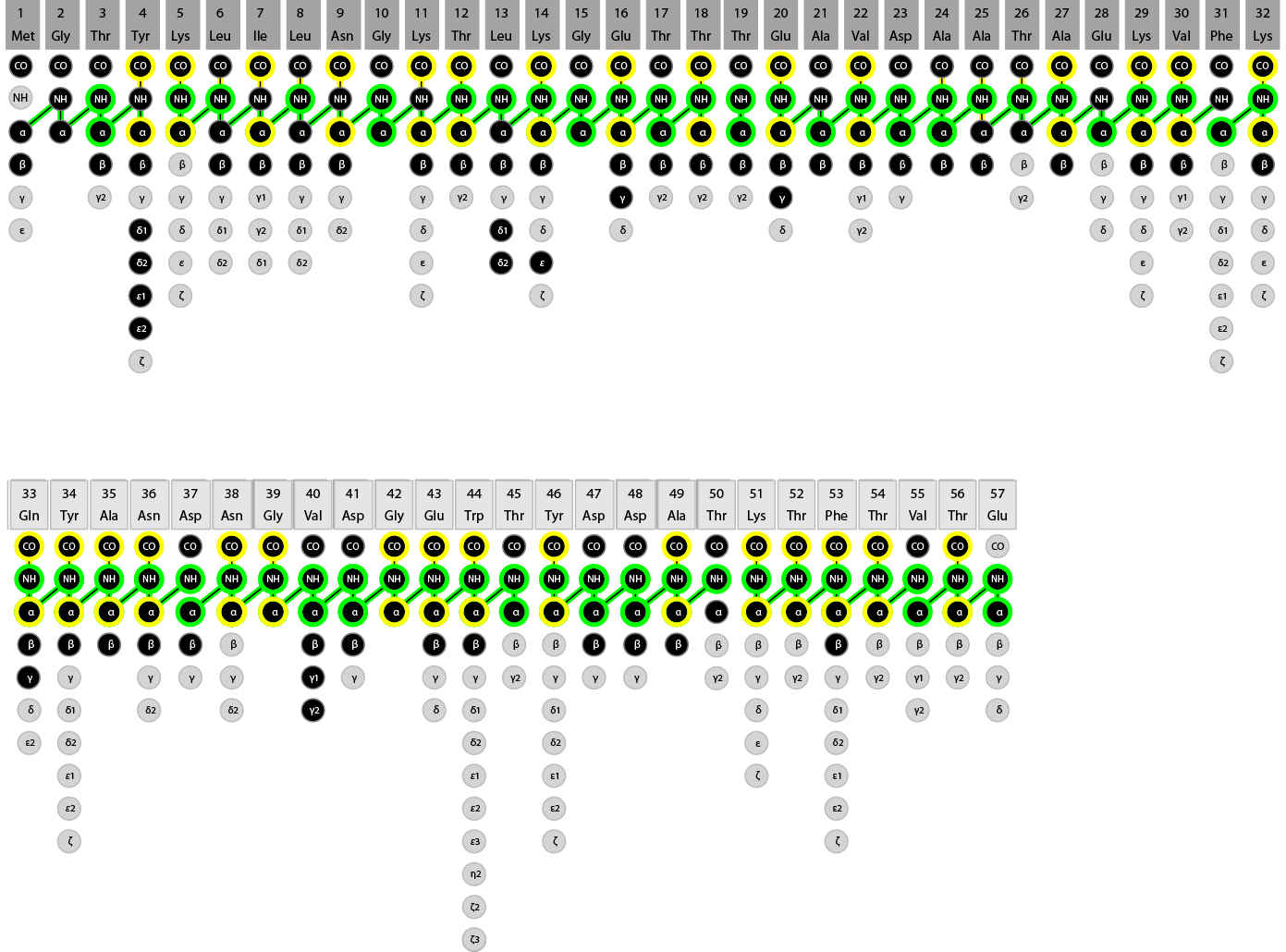


**Supplementary Figure S7. Backbone and side-chain resonance assignments of GB1 in living *E. coli* cells.** Filled circles in black and light grey indicate assigned and unassigned atoms, respectively. Atoms assigned in the HCACO and HNCA spectra are marked by yellow and green circles, respectively. Intra- and sequential HNCA connectivities are indicated by green lines. Drawing made with the software CcpNmr Analysis.


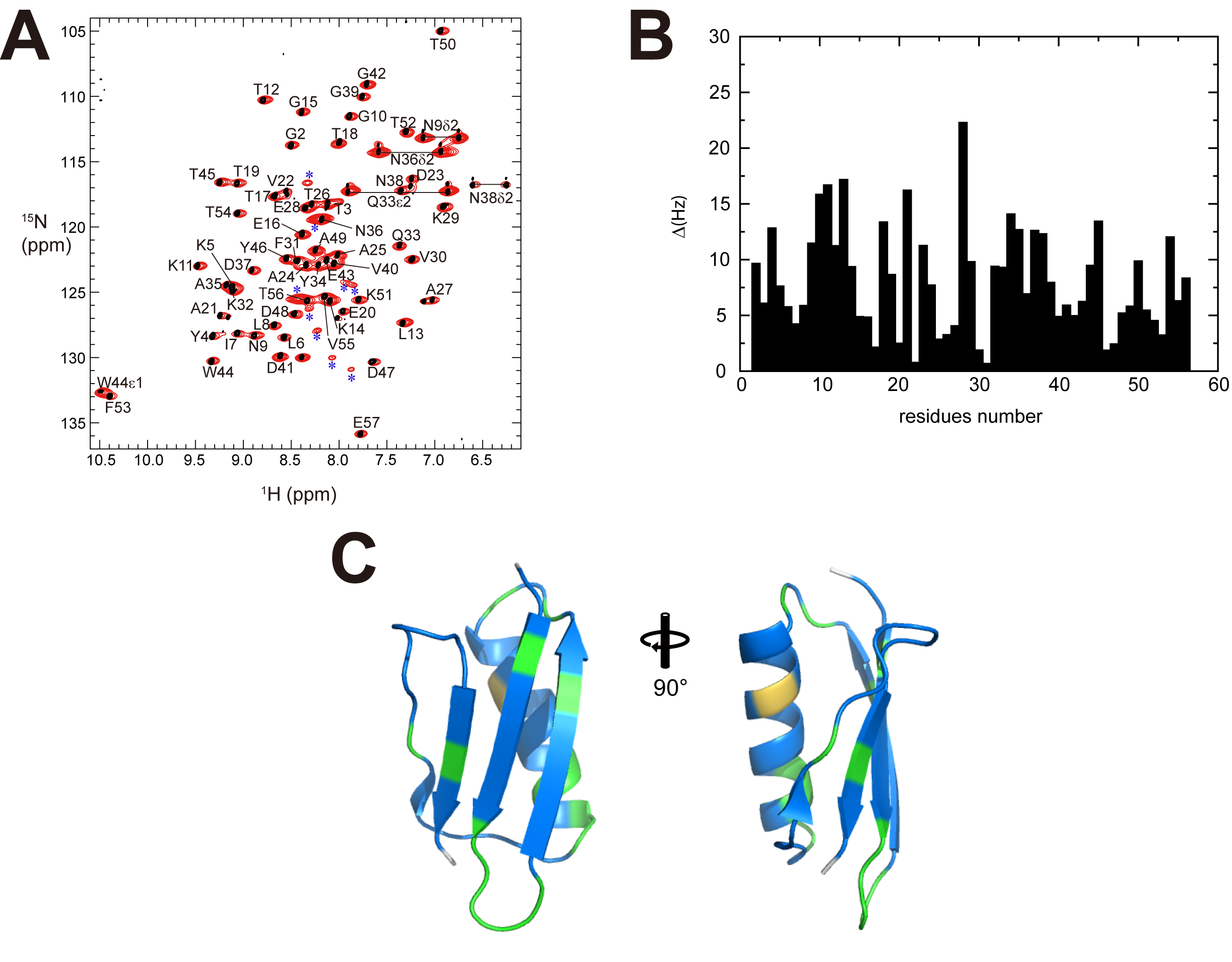


**Supplementary Figure S8. Chemical shift differences of backbone ^1^H^N^ and ^15^N nuclei of GB1 between in *E.coli* cells and *in vitro* conditions.** (*A*) The superposition of in-cell and *in vitro* spectra. Asterisks show background signals derived from cell’s compounds. (*B*) Chemical shift differences *Δ* = (*Δ*_H_^2^ + *Δ*_N_^2^)^1/2^, where *Δ*_H_ and *Δ*_N_ are the differences in Hz for the backbone amide ^1^H and ^15^N chemical shifts between the two conditions. 1 ppm corresponds to 600.13 Hz for ^1^H and 60.81 Hz for ^15^N. (*C*) Chemical shift differences shown by light orange (*Δ *> 20 Hz), green (*Δ = *20-10 Hz), blue (*Δ *< 10Hz), and white (unassigned) on a ribbon model of the NMR structure of GB1 (PDB-ID:2N9K).

**
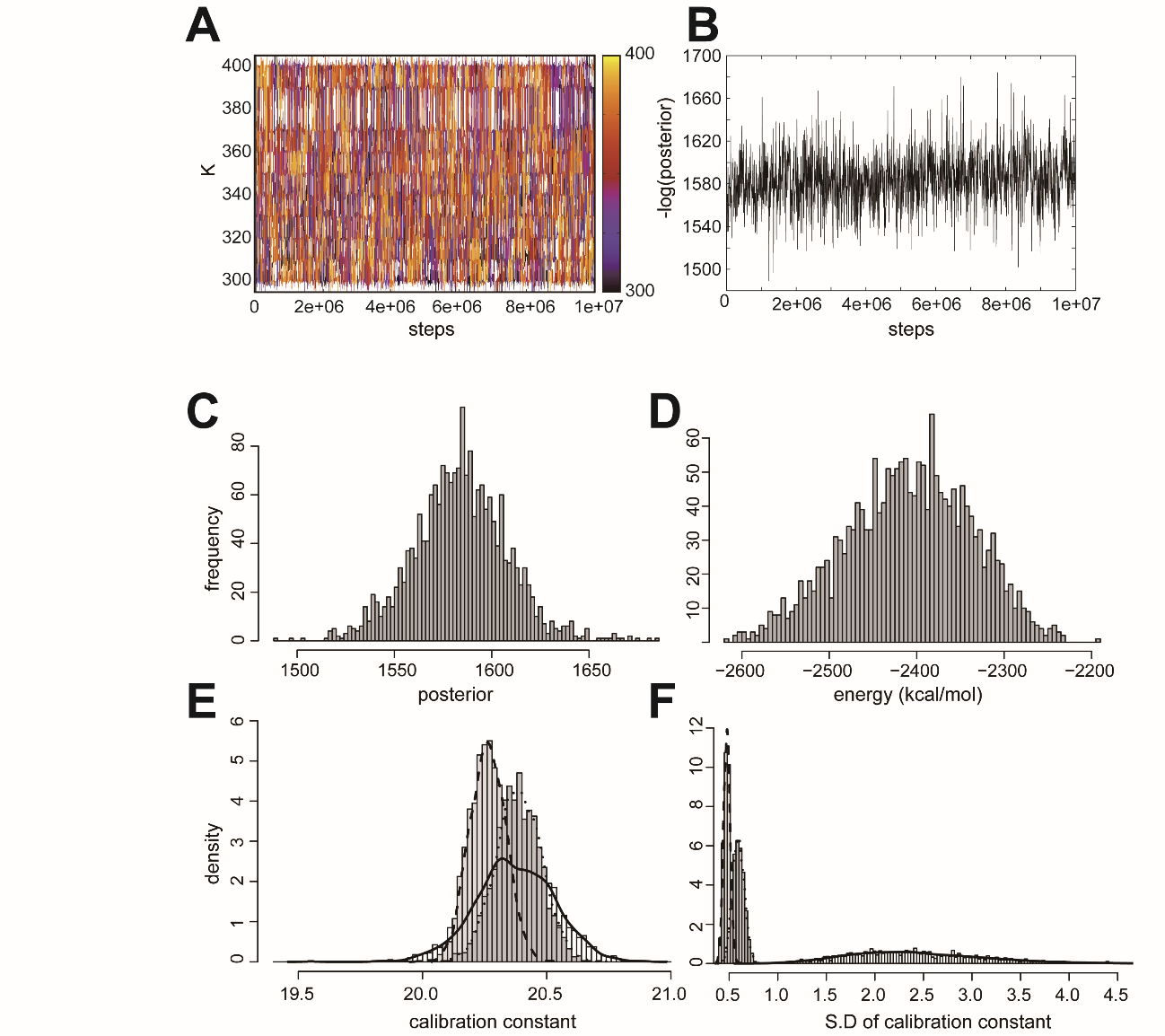
**

**Supplementary Figure S9. CYBAY statistics for GB1 in *E. coli* cells.** (*A*) Fraction of temperatures as a function of the step number. Structures from 10 calculations (replicas) at temperatures of 300, 310, 320, 330, 340, 350, 360, 370, 390, and 400 K were exchanged every 10000 Monte Carlo (MC) steps. The line colors indicate the initial temperatures of each replica corresponding to the vertical color panel, demonstrating that all replicas exchanged adequately and passed through all temperatures in the whole process. (*B*) Negative logarithm of the posterior. Its values are on average almost stable, suggesting that the number of MC steps is sufficient for sampling. (*C*) Posterior distribution of the in-cell GB1 conformers. (*D*) Potential energy distribution of the in-cell GB1 conformations. (*E*) Distributions of the calibration constants computed by CYBAY for ^13^C/^13^C-separated (bold line), ^13^C-separated (dashed line), and ^15^N-separated (dotted line) NOESY spectra (*F*) Corresponding distributions of the standard deviation of the calibration constants. The distributions reflect the quality and quantity of the experimental data more directly than those of the structures. In particular, the distributions of the calibration constant and its standard deviation for the ^13^C/^13^C-separated NOESY were broader than for the ^15^N-separated and ^13^C-separated NOESYs, presumably due to the smaller number of peaks, and the concomitant smaller information content.

**
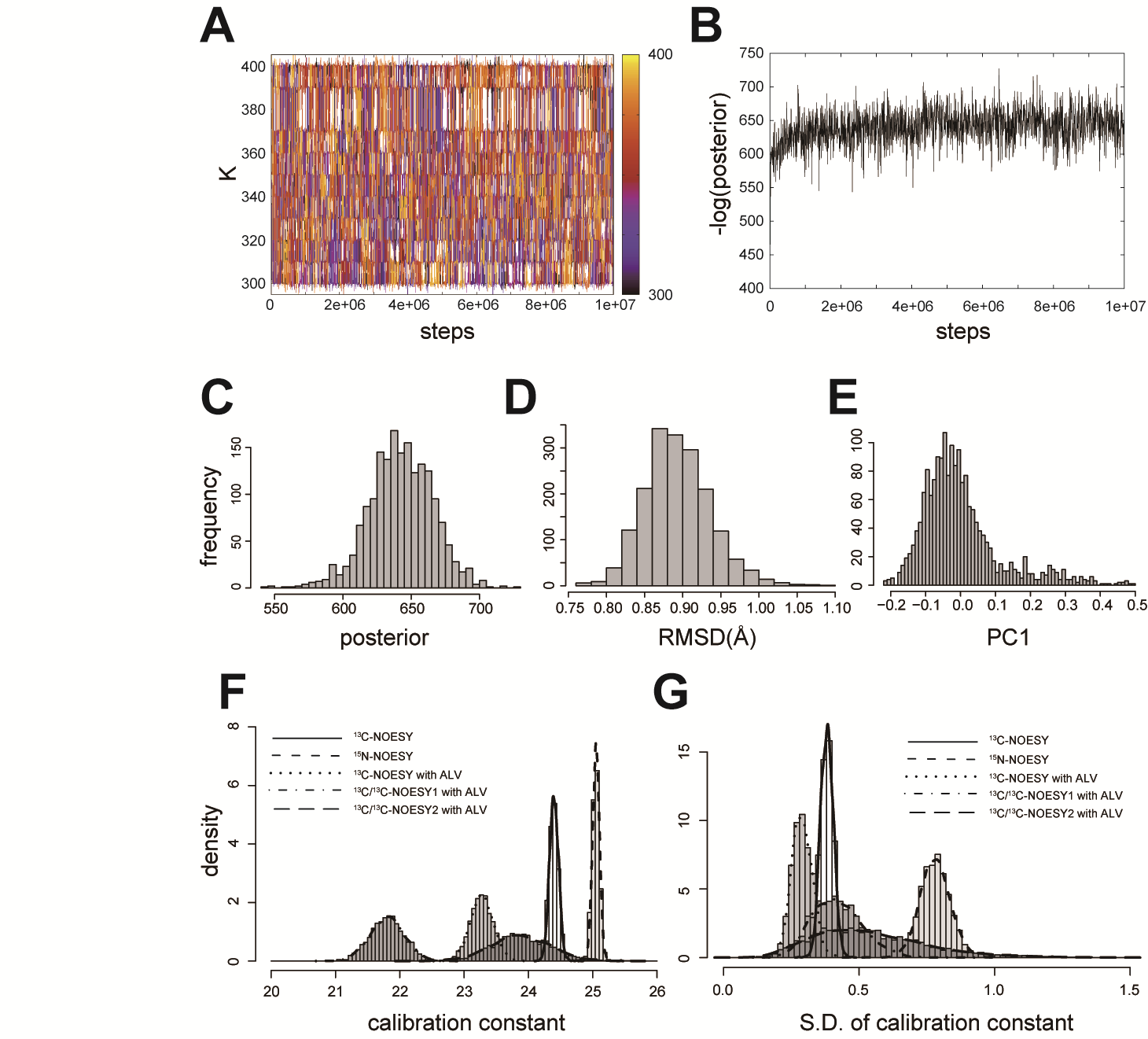
**

**Supplementary Figure S10. CYBAY statistics for TTHA1718 in *E.coli* cells.** (*A*) Trace of step evolutions of fraction of temperatures. The 10 independent calculations, replicas, were exchanged at 300 to 400 K in 10000 Monte Carlo (MC) steps. The line colors show the initial temperatures of each replica corresponding to the vertical color panel. (*B*) Negative logarithm of the posterior distribution at the lowest temperature. (*C*) Posterior distribution of the in-cell TTHA1718 conformations. (*D*) RMSD distribution of the in-cell TTHA1718 conformations to the *in vitro*. (*E*) Distribution of the first principal component of Principal Component Analysis. (*F*) Distributions of the calibration constants of five NOESY spectra. (*G*) Corresponding distributions of the standard deviation of the calibration constants. The ^13^C/^13^C-separated NOESY spectrum with methyl-selectively labeled Ala/Leu/Val (ALV) was divided into two regions for NMR data processing, and applied as different inputs into CYBAY. Those are indicated as ^13^C/^13^C NOESY1 and 2 with ALV in Figure *F* and *G*.


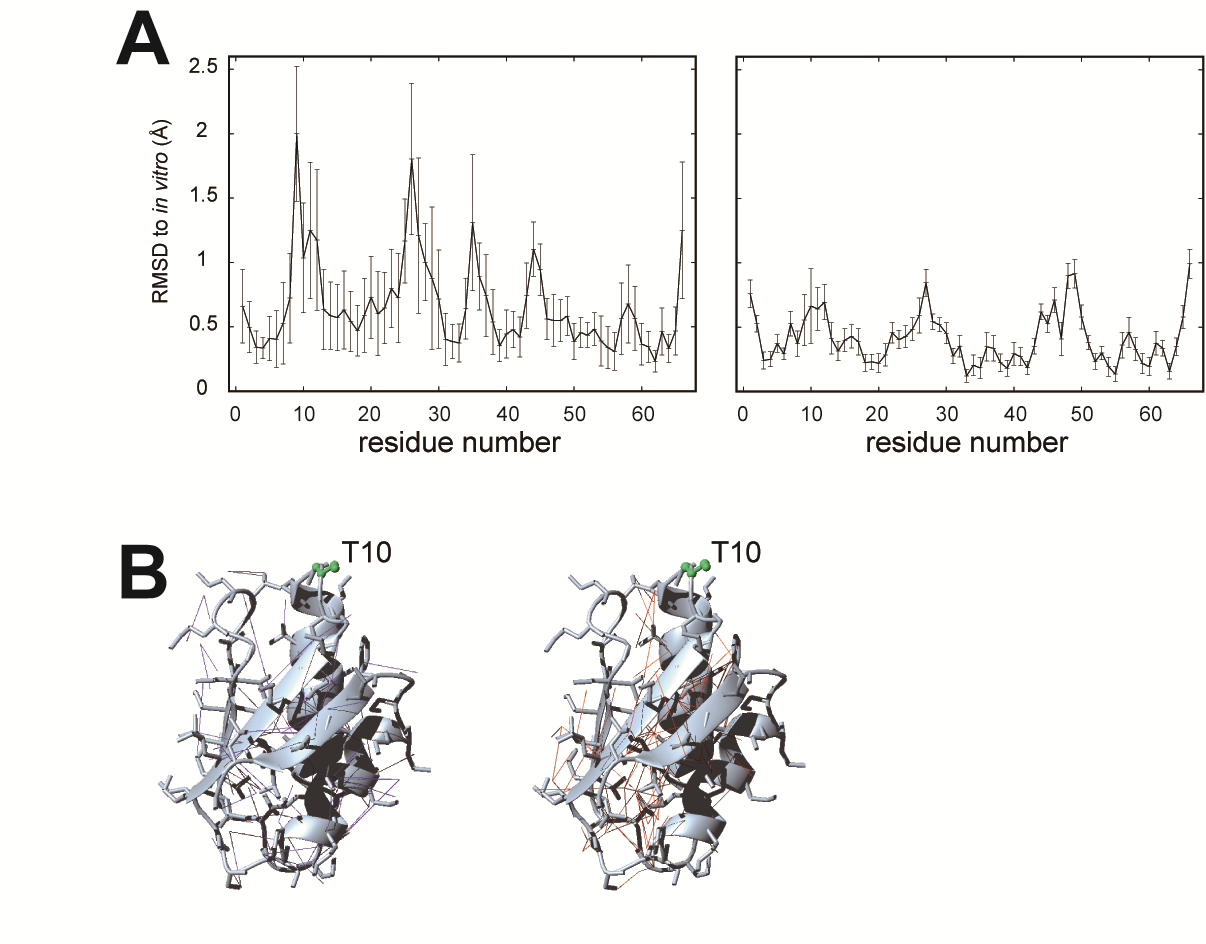


**Supplementary Figure S11. RMSDs for residues of TTHA1718 to the *in vitro* structures, and medium and long range distance restrains.** (*A*) C^α^ RMSDs of the previously reported (left) and the re-calculated structures (right) for residues of TTHA1718 to the *in vitro* structures**.** Error bars show standard deviation of RMSD in 20 of the previous structures and 1800 conformers derived from the Bayesian calculation. (*B*) Distance restraints additionally obtained by the QME processing and FLYA automatic chemical shift assignment. Medium range (blue lines in the left panel) and long range (red lines in the right panel) restraints are represented in the white ribbon models. The green ball-and-stick models show Thr 10, which is one of the putative metal binding residues.

**Supplementary Table S1.** 3D NMR spectra measured for GB1 in living cells

| Experiments | Sampling space (complex)^b^  (*t_1_*) × (*t_2_*) | Sampled complex points^c^ (%) | NS^d^ | Duration^e^ | Combined data sets^f^ |
| --- | --- | --- | --- | --- | --- |
| HNCA | 32 (^13^C) × 22 (^15^N) | 176 (25%) | 8 | 95 min | 2 |
| HN(CO)CA | 48 (^13^C) × 22 (^15^N) | 264 (25 %) | 8 | 140 min | 2 |
| CBCANH^a^ | 48 (^13^C) × 22 (^15^N) | 264 (25 %) | 8 | 140 min | 2 |
| CBCA(CO)NH | 48 (^13^C) × 22 (^15^N) | 264 (25 %) | 8 | 140 min | 2 |
| HN(CA)CO^a^ | 32 (^13^C) × 22 (^15^N) | 176 (25 %) | 16 | 190 min | 2 |
| HNCO | 48 (^13^C) × 22 (^15^N) | 264 (25 %) | 8 | 140 min | 2 |
| HCACO | 26 (^13^C^a^) × 32 (^13^C’) | 208 (25 %) | 8 | 110 min | 2 |
| HBHA(CBCACO)NH | 32 (^1^H) × 22 (^15^N) | 176 (25%) | 8 | 95 min | 2 |
| CC(CO)NH | 32 (^13^C) × 22 (^15^N) | 176 (25%) | 8 | 95 min | 2 |
| H(CCCO)NH | 32 (^1^H) × 22 (^15^N) | 176 (25%) | 8 | 95 min | 2 |
| HCCH-COSY | 64 (^1^H) × 32 (^13^C) | 256 (12.5%) | 8 | 140 min | 2 |
| HCCH-TOCSY | 64 (^1^H) × 22 (^13^C) | 352 (25 %) | 8 | 190 min | 2 |
| ^15^N-separated NOESY-HSQC | 48 (^1^H) × 12 (^15^N) | 144 (25 %) | 16 | 150 min | 1 |
| ^13^C-separated NOESY-HSQC | 48 (^1^H) × 12 (^13^C) | 288 (50 %) | 16 | 310 min | 1 |
| ^13^C/^13^C-separated HMQC-NOE-HMQC | 32 (^13^C) ×16 (^13^C) | 128 (25 %) | 16 | 140 min | 1 |

^a^ These experiments were practically not used for resonance assignment because of insufficient signal to noise ratio.

^b^ Conventional regularly spaced grid for indirectly observed dimensions (*t_1_*, *t_2_*).

^c^ Complex points selected in a pseudo-random fashion.

^d^ Number of scans per FID.

^e^ Measurement time.

^f^ Number of 3D data sets combined for processing.

**Supplementary Table S2.** NMR structure statistics of GB1 with in-cell NMR data

| Quantity^a^ | *in vitro*^b^ | manual^c^ | FLYA^d^ | CYBAY^e^ |
| --- | --- | --- | --- | --- |
| Assigned ^1^H/^13^C/^15^N chemical shifts | 238/182/61 | 159/165/57 | 207/217/58 | 207/217/58 |
| Distance restraints^f^ | 701/318/597 | 162/42/78 | 233/49/108 | 233/49/108 |
| Restrained hydrogen bonds | – | – | – | – |
| Dihedral angle restraints (*ϕ*/*ψ*) | 102 | 102 | 106 | 106 |
| Max. distance restraint violation (Å) | 0.10 ± 0.00 | 0.12 ± 0.01 | 0.16 ± 0.15 | — |
| Max. dihedral angle violation (º) | 2.2 ± 0.4 | 2.9 ± 0.8 | 2.7 ± 0.7 | 2.5 ± 0.6 |
| Deviations from idealized geometry: |  |  |  |  |
| Bond lengths (Å) | 0.0139±0.0001 | 0.0139±0.0001 | 0.0139±0.0001 | 0.0140±0.001 |
| Bond angles (º) | 1.72 ± 0.04 | 1.78 ± 0.07 | 1.78 ± 0.07 | 1.73 ± 0.03 |
| AMBER energy (kcal/mol) | –2531 ± 62 | –2062 ± 107 | –2103 ± 77 | –2408 ± 67 |
| AMBER vdW energy (kcal/mol) | –178 ± 3 | –120 ± 10 | –136 ± 10 | –186 ± 10 |
| Ramachandran plot statistics^g^ (%) | 88/12/0/0 | 89/11/0/0 | 88/12/0/0 | 87/13/0/0 |
| Backbone RMSD (Å) | 0.21 ± 0.03 | 1.74 ± 0.55 | 0.61 ± 0.18 | 0.49 ± 0.11 |
| All heavy atom RMSD (Å) | 0.47 ± 0.03 | 2.39 ± 0.53 | 1.15 ± 0.22 | 0.71 ± 0.10 |
| Backbone RMSD to *in vitro* (Å)^h^ | – | 1.81 | 1.45 | 1.02 |
| All heavy atom RMSD to *in vitro* (Å)^h^ | – | 2.52 | 2.06 | 1.73 |

^a^Where applicable, the average value and the standard deviation over the 20 energy-refined conformers that represent the NMR structure are given.

^b^Statistics for *in vitro* GB1 structures. In the number of the chemical shift assignments, C’ atoms are not assigned and included in those numbers.

^c^Structure calculated without manually analyzed hydrogen bond distance restraints.

^d^Structure calculated with automatically assigned chemical shifts by FLYA, and no hydrogen bond restraints.

^e^Structure calculated with CYBAY refinement, FLYA chemical shifts, and no hydrogen bond restraints. For comparison with other data, the statistics were calculated for the 20 highest posterior structures.

^f^Short/medium/long-range distance restraints derived from NOESY spectra.

^g^Percentage of residues in the most favored/additionally allowed/generously allowed/disallowed regions of the Ramachandran plot according the program PROCHECK.

^h^RMSD between the mean structure of the ensemble and the *in vitro* mean structure.

**Supplementary Table S3.** NMR structure statistics of TTHA1718 with in-cell NMR data

| Quantity^a^ | reported^b^ | no hb^c^ | QME^d^ | FLYA^e^ | CYBAY^f^ |
| --- | --- | --- | --- | --- | --- |
| Assigned ^1^H/^13^C/^15^N chemical shifts | 70/207/62 | 70/207/62 | 70/207/62 | 74/267/62 | 74/267/62 |
| Distance restraints^g^ | 179/24/89 | 180/28/83 | 231/23/75 | 322/98/188 | 322/98/188 |
| Restrained hydrogen bonds | 23 | — | — | — | — |
| Dihedral angle restraints (*ϕ*/*ψ*) | 90 | 90 | 90 | 114 | 114 |
| Max. distance restraint violation (Å) | 0.13 ± 0.01 | 0.14 ± 0.01 | 0.14 ± 0.01 | 0.13 ± 0.01 | — |
| Max. dihedral angle violation (º) | 2.2 ± 0.5 | 2.2 ± 0.7 | 2.6 ± 0.8 | 2.8 ± 0.4 | 8.6 ± 1.8 |
| Deviations from idealized geometry: |  |  |  |  |  |
| Bond lengths (Å) | 0.0135±0.0001 | 0.0134±0.0001 | 0.0175±0.0006 | 0.0133±0.0001 | 0.0689±0.0217 |
| Bond angles (º) | 1.75 ± 0.04 | 1.76 ± 0.04 | 2.55 ± 0.75 | 1.89 ± 0.05 | 1.83 ± 0.07 |
| AMBER energy (kcal/mol) | -2496 ± 100 | -2446 ± 110 | -2316 ± 74 | -2503 ± 44 | -2638 ± 62 |
| AMBER vdW energy (kcal/mol) | -162 ± 13 | -160 ± 11 | -148 ± 7 | -165 ± 10 | -214 ± 8 |
| Ramachandran plot statistics^h^ (%) | 92/7/1/0 | 90/9/1/0 | 87/12/1/0 | 88/12/0/0 | 88/12/0/0 |
| Backbone RMSD (Å) | 0.96 ± 0.20 | 1.25 ± 0.21 | 0.95 ± 0.19 | 0.64 ± 0.11 | 0.40 ± 0.06 |
| All heavy atom RMSD (Å) | 1.53 ± 0.21 | 1.86 ± 0.17 | 1.48 ± 0.20 | 1.08 ± 0.10 | 0.67 ± 0.08 |
| Backbone RMSD to *in vitro* (Å)^i^ | 1.16 | 1.98 | 1.40 | 0.87 | 0.82 |
| All heavy atom RMSD to *in vitro* (Å)^i^ | 1.87 | 2.50 | 2.02 | 1.35 | 1.22 |

^a^Where applicable, the average value and the standard deviation over the 20 energy-refined conformers that represent the NMR structure are given.

^b^Statistics for previously reported in-cell TTHA1718 structures.

^c^Statistics for TTHA1718 calculated without manually analysed hydrogen bond distance restraints.

^d^Statistics for TTHA1718 calculated with ^13^C-separated, ^15^N-separated NOESY and 13C/13C-separated NOESY spectra reconstructed by QME, and no hydrogen bond restraints.

^e^Statistics for TTHA1718 calculated with the QME-processed spectra and additional chemicals shifts automatically assigned by FLYA, and no hydrogen bond restraints.

^f^Statistics for TTHA1718 calculated with the CYBAY refinement, QME-processed spectra and FLYA chemicals shifts, and no hydrogen bond restraints. In order to compare other data, the statistics were calculated in the 20 highest posterior structures.

^g^Short/medium/long-range distance restraints derived from NOESY spectra.

^h^Percentage of residues in the most favored/additionally allowed/generously allowed/disallowed regions of the Ramachandran plot according the program PROCHECK.

^i^RMSD between the mean structure of the ensemble and the *in vitro* mean structure.
